# Iranian Clinical Nurses’ Activities for Self-Directed Learning: A Qualitative Study

**DOI:** 10.5539/gjhs.v8n5p48

**Published:** 2015-08-31

**Authors:** Shahrzad Ghiyasvandian, Morteza Malekian, Mohammad Ali Cheraghi

**Affiliations:** 1 School of Nursing and Midwifery, Tehran University of Medical Sciences, Tehran, Iran; 2 School of Nursing and Midwifery, Tehran University of Medical Sciences, International Campus (TUMS-IC), Tehran, Iran

**Keywords:** clinical nurses, self-directed learning, lifelong learning, qualitative study, Iran

## Abstract

**Background:**

Clinical nurses need lifelong learning skills for responding to the rapid changes of clinical settings. One of the best strategies for lifelong learning is self-directed learning. The aim of this study was to explore Iranian clinical nurses’ activities for self-directed learning.

**Methods:**

In this qualitative study, 23 semi-structured personal interviews were conducted with nineteen clinical nurses working in all four hospitals affiliated to Isfahan Social Security Organization, Isfahan, Iran. Study data were analyzed by using the content analysis approach. The study was conducted from June 2013 to October 2014.

**Findings:**

Study participants’ activities for self-directed learning fell into two main categories of striving for knowledge acquisition and striving for skill development. The main theme of the study was ‘Revising personal performance based on intellectual-experiential activities’.

**Conclusions:**

Study findings suggest that Iranian clinical nurses continually revise their personal performance by performing self-directed intellectual and experiential activities to acquire expertise. The process of acquiring expertise is a linear process which includes two key steps of knowledge acquisition and knowledge development. In order to acquire and advance their knowledge, nurses perform mental learning activities such as sensory perception, self-evaluation, and suspended judgment step-by-step. Moreover, they develop their skills through doing activities like apprenticeship, masterly performance, and self-regulation. The absolute prerequisite to expertise acquisition is that a nurse needs to follow these two steps in a sequential manner.

## 1. Introduction

Rapid scientific advances have significantly decreased the half life of medical sciences (Gyawali, Jauhari, Shankar, Saha, & Ahmad, 2011) and posed big challenges to healthcare systems and fields, including nursing (Yang & Jiang, 2014). Accordingly, nurses are expected to have certain kinds of learning skills for managing rapid changes in healthcare settings. Moreover, as nursing is an applied science, nurses need to learn how to transfer their knowledge to practice. Papathanasiou, Tsaras and Sarafis (2014) also noted that nurses’ active involvement in their own learning is an absolute prerequisite to lifelong learning.

One of the good strategies for lifelong learning is self-directed learning (Fisher & King, 2010). The concept of self-directed learning (SDL) originates from the Adult Learning Theory. This theory suggests that adults are pragmatic and problem-focused individuals whose learning is mainly affected by experiential rather than passive approaches (Roberson, 2011). SDL is a process in which learners actively participate in identifying their own learning needs, setting learning goals, allocating resources, developing and implementing appropriate strategies and plans, and evaluating learning outcomes either independently or with others’ help (Knowles, Holton, & Swanson, 2008). It could help nurses identify their learning needs, plan for fulfilling the identified needs, and turn into independent experts (Montin & Koivisto, 2014). The positive outcomes of SDL include, but not limited to, greater self-control, self-confidence, autonomy, and lifelong learning skills (O’Shea, 2003).

Many studies have been conducted so far in different countries on the concept of SDL, readiness for it, and its effectiveness. The results of an interventional study conducted by Saha (2006) showed that compared with traditional approaches, student-centered learning significantly increased Indonesian students’ SDL readiness as well as their accountability for learning. Nokdee (2007) also conducted a qualitative study to explore Thai nurses’ experiences of SDL and reported that the main attributes of SDL were greater independence in and responsibility for learning, enhanced problem-solving ability, and improved effectiveness of learning. Moreover, she found that nurses learn through implementing nursing procedures and interacting with patients, colleagues, and physicians. O’Shea (2003) conducted a literature review on SDL in nursing education and found that this concept had different domains including the definition and the entity of SDL, SDL readiness, SDL styles, SDL facilitators, and SDL benefits. O’Shea (2003) also noted that despite great efforts for clarifying the concept of SDL in the last three decades, a comprehensive understanding of the concept is still lacking. Cazan and Schiopca (2014) also conducted a study to examine the relationships among SDL, personality traits, and academic achievements and found that SDL is significantly correlated with academic achievements and personality traits of extraversion and openness. Safavi, Schooshtarizadeh, Mahmoodi and Yarmohammadian (2010) also investigated SDL readiness and styles among 178 Iranian nursing students and reported that 34.3%, 32.6%, and 33.1% of the participating students had respectively high, moderate, and low SDL readiness. However, Karshki, Mohammadzadeh Ghaser, Taghizadeh and Garavand (2013) reported that nursing students’ SDL readiness was great.

Despite the wealth of studies on SDL, this concept has not yet been completely clarified and hence, many ambiguities surround it. For instance, terms such as self-planned learning, autonomous learning, project-based learning, independent study, self-teaching, and autodidaxy are used for addressing SDL (Murad & Verkey, 2008). Moreover, most of the previous studies were conducted on nursing students and hence, few studies have dealt with clinical nurses’ SDL. On the other hand, learning is greatly affected by culture and social context and therefore, studying SDL in different cultures is needed (Ahmad & Majid, 2010). Nonetheless, few studies have been conducted so far in this area in our country, Iran. This study was designed and done to reduce these gaps. The aim of the study was to explore Iranian clinical nurses’ activities for SDL.

## 2. Methods

The qualitative content analysis approach was used in this study. This approach deals with inductively developing categories and interpreting textual or verbal data and is used when there is limited knowledge about a certain phenomenon (Cho & Lee, 2014).

The study setting was all four hospitals affiliated to Isfahan Social Security Organization, Isfahan, Iran. The hospitals were located in Isfahan and Najafabad, Iran. All these hospitals included medical-surgical, pediatric, midwifery, and intensive care units at the time of the study. Purposive sampling technique was used for recruiting nurses who had in-depth information about SDL. The inclusion criteria were having a minimum of one-year work experience in clinical nursing, actively participating in continuing education programs, and either holding Bachelor’s or Master’s degrees or being a postgraduate student in nursing. We kept on sampling until reaching data saturation, i.e. until obtaining no new information from the data (Polit & Beck, 2014).

### 2.1 Data Collection

Study data were collected by carrying out semi-structured personal interviews organized according to participants’ preferences. Interviews were conducted in a private room located in participants’ workplaces. The length of the interviews ranged from 35 to 115 minutes with a mean of 56 minutes. As immediate documentation of the data is a prerequisite to successful data analysis (Adib-Hajbagheri, Parvizi, & Salsaly, 2013), interviews were immediately transcribed verbatim. The interview opening questions were: Would you please explain your experiences of clinical learning? What do you do for advancing your knowledge and developing expertise? What factors have contributed to your clinical learning? What activities do you perform for promoting your clinical learning? In addition, we employed probing questions for clarifying participants’ responses. We reached data saturation after conducting 23 interviews with nineteen participants.

### 2.2 Data Analysis

Study data were analyzed by using the conventional content analysis approach (Graneheim & Lundeman, 2004). This approach is used when the aim is to describe a poorly-known phenomenon. We avoided using predetermined categories and instead let categories emerge from the data (Elo & Kyngas, 2008). Initially, we listened to the interviews and reviewed interview transcripts for several times to immerse in the data and acquire a general understanding of them. This technique helped us identify the meaning units. Then, we read each interview transcript line-by-line and extracted and coded all words, sentences, and paragraphs containing meaning units. The data and the generated codes were constantly compared. Accordingly, codes were categorized according to their similarities. Primary categories were in turn combined to form more abstract categories. Finally, the study theme was generated (Krippendorff, 2013).

### 2.3 Ethical Considerations

This study was primarily approved by the Research Council and the Ethics Committee of Tehran University of Medical Sciences, Tehran, Iran. Before starting the study, we obtained necessary permissions from the administrators of the study setting. Moreover, study participants were informed about the aim of the study, the process of interview, the confidentiality of their information, and the voluntariness of participation in the study. Finally, informed consent was obtained from them.

### 2.4 Trustworthiness

We used the four criteria of credibility, confirmability, dependability, and transferability for ensuring the trustworthiness of the data and the findings (Elo et al., 2014). The credibility of the findings was established by using techniques such as member-checking, prolonged engagement with the study (about two years), and close relationship with the participants. On the other hand, the external peer-checking method was used for enhancing the confirmability of the findings. Accordingly, pieces of the data and the corresponding findings were sent to an experienced qualitative researcher and two PhD students in nursing. They confirmed the soundness of the analyses and provided useful advice. The dependability of the findings was also ensured by exactly documenting all steps of the study. In addition, the maximum variation sampling technique was adopted for enhancing the transferability of the findings.

## 3. Findings

Nineteen nurses (thirteen staff nurses, three head-nurses, two supervisors, and one matron) participated in the study. The nurses held either Bachelor’s (seven individuals) or Master’s degrees (twelve individuals) in nursing. Nine participants were male and ten were female. Participants aged from 25 to 44 with a mean of 32 years. The mean of participants’ work experience was eight years with a range of three to nineteen years.

Study participants’ activities for SDL were grouped into two main categories including striving for knowledge acquisition and striving for skill development. The main theme of the study was ‘Revising personal performance based on intellectual-experiential activities (Table 1). This theme implies that nurses constantly monitor their performance in order to develop their expertise and revise their performance by doing intellectual activities for acquiring knowledge as well as experiential activities for developing their skills. The two main categories of the study are explained in what follows.

**Table 1:** The main theme, categories, and sub-categories of the study

Sub-category	Category	Main Category	Main Theme
Careful observation			
Active listening	Sensory perceptions		
		
Desire for questioning			
Reflection on performance	Self-evaluation	Striving for knowledge acquisition	Revising personal performance based on intellectual-experiential activities

Early risk identification	Suspended judgment		
Hypothesis making			

Learning from an expert colleague	Apprenticeship		
Learning support			
		
Role-modeling	Masterly performance		
Anxiety management		Striving for skill development	
	
Maintaining a caring attitude			
Having a subjective care plan	Self-regulation		
Practicing as a prerequisite to expertise			

### 3.1 Striving for Knowledge Acquisition

This main category encompasses activities which nurses performed in order to collect accurate and credible information for analyzing and evaluating their own performance. The three categories of this main category were sensory perceptions, self-evaluation, and suspended judgment.

#### 3.1.1 Sensory Perceptions

Our participants used sensory stimulations to stimulate their senses and perceive events. Sensory perception is an activity through which a nurse sees, listens, and assesses events from different perspectives. This category included two sub-categories which are explained below.

3.1.1.1 Careful Observation

One of nurses’ activities for SDL was learning through observing patients, colleagues, and physicians. According to our participants, observing the signs and the symptoms of diseases helped them confirm and internalize their already-acquired knowledge. A female nurse having a four-year work experience said,

*Observation has helped me to promote my learning. I have learned a lot through caring for patients with different diagnoses and rare diseases. I validated my knowledge by the signs and the symptoms observed in patients. This strategy helped me learn and internalize the signs and the symptoms of diseases (a female nurse (P. 8)*.

Moreover, observing colleagues and physicians’ clinical practice had helped our participants promote their learning, advance their knowledge, and correct their misconceptions. A male nurse with a work experience of fifteen years expressed,

*Despite having a wealth of academic information, many skills that I had learned in skill lab were not useful to me until observing experienced colleagues and physicians implementing nursing procedures such as dressing. Observation was very helpful to me. I reviewed the steps of the procedures while observing [others doing] them (P. 12)*.

3.1.1.2 Active Listening

Our participants’ another activity for SDL was active listening. As routine activities for SDL, they processed, analyzed, and memorized what they had listened to. According to them, obtaining and analyzing different useful ideas and information during nursing and medical rounds help nurses revise and advance their knowledge. A female nurse having a work experience of five years mentioned,

*We had a patient in our ward who needed central venous pressure (CVP) monitoring. During a nursing round, one of our colleagues said that for CVP monitoring, the head of bed should be elevated 30 degrees. Other colleagues also agreed. However, I was doubtful and referred to a textbook where I found that patient should be placed in flat position during CVP monitoring. This fact was imprinted on my and my colleagues’ memories (P. 7)*.

Moreover, active listening to patients was another way for gathering information. A male nurse having a sixteen-year work experience highlighted,

*I have learned to listen to patients because by using this strategy, I can both establish a therapeutic communication with them and get familiar with the subjective symptoms of their diseases (P. 1)*.

#### 3.1.2 Self-Evaluation

Self-evaluation is an intentional and masterly activity which necessitates performance evaluation as well as judgment about the effectiveness of one’s own performance for finding more effective alternatives. During self-evaluation, our participants assessed their own previous experiences and understood why and how the events happened. The two sub-categories of this category were desire for questioning and reflection on performance.

3.1.2.1 Desire for Questioning

When being faced with work-related problems, our participants strived to find their underlying causes, challenge the work routines, and review their own knowledge and expertise in order to promote their learning. A male nurse who had a five-year work experience mentioned,

*During my daily practice, I spent a greater deal of time and thought more carefully to understand the underlying causes of patients’ problems (P. 17)*.

A male nurse with a work experience of eighteen years also noted,

Whenever I was allocated to a new ward, I understood that the staffs of that ward do their tasks based on a same routine. Perhaps, they had a convincing reason for such routine practice which was, ‘Routines are more convenient’. However, I refrained following routines and asked myself, ‘Can’t we do this task on any other ways?’

3.1.2.2 Reflection on Performance

Our nurses raised probing questions about their own performance and experiences, referred to different sources to find answers to their questions, compared their own performance with standard clinical guidelines, and accordingly, chose new styles and guidelines for their clinical practice. A male nurse having a fifteen-year work experience highlighted,

*I have understood that each experience can be reflected on and such reflection can broaden that experience. Consequently, I usually question my experience to be obliged to think about it more carefully. For instance, I admitted a patient who had just undergone an appendectomy. He had a constant pain. I called the surgeon and administered analgesic for several times. However, the pain was persistent. Finally, the patient developed hypotension and was transferred again to the operating room. The diagnosis was pyloric rupture. I questioned myself to understand what type of care I had to provide to this patient (P. 12)*.

#### 3.1.3 Suspended Judgment

The third category of the first main category was suspended judgment. This category implies using thinking and logic in clinical situations. Suspended judgment is a vital skill for identifying potential risks, establishing early diagnosis by using one’s own experience, and evaluating clinical decisions. This category consisted of two sub-categories which are explained below.

3.1.3.1 Early Risk Identification

Most of our participants mentioned that they employed their cognitive skills and professional knowledge to identify probable risks to patients’ health. This screening strategy was implemented in several steps including recognizing the signs and the symptoms of the risk, predicting its probability, making clinical decisions, and developing a care plan. A male head-nurse with a twelve-year work experience expressed,

*We had a patient in our unit who had experienced an injury to his seventh cervical vertebra (C7). At shift turnover, I said [to my colleagues] that you need to be ready because the edema will soon progress from C7 toward C1 and the medulla, suppressing patient’s respiratory center and causing apnea. I put a ventilator standby at patient’s bedside and explained the care plan for the colleagues. Predictions were accurate and when I returned to the unit the next morning, the patient had been attached to the ventilator (P. 15)*.

3.1.3.2 Hypothesis Making

Our participants performed different mental activities such as data collection, hypothesis making, and hypothesis testing to reach an accurate nursing diagnosis. They highlighted that the knowledge which they had gained in university was not helpful to them for making and testing hypotheses. Instead, they had learned these skills through referring to other sources such as their own experiences as well as experienced colleagues and physicians. A male nurse who had a four-year work experience said,

*I always try to make some hypotheses based on the signs and the symptoms experienced by a patient. For instance, when a patient has tachypnea, I ask myself whether this patient is suffering from oxygen saturation disturbances, or she/he needs a chest X-ray assessment, or this sign is due to other problems such as pain (P. 18)*.

### 3.2 Striving for Skill Development

The second main category of the study was striving for skill development. This main category covered three categories including apprenticeship, masterly performance, and self-regulation which are explained below.

#### 3.2.1 Apprenticeship

Apprenticeship is a teaching process in which a junior nurse voluntarily chooses to be supervised and trained by an expert colleague. The two sub-categories of this category were learning from an expert colleague and learning support.

3.2.1.1 Learning From an Expert Colleague

Our participants considered the presence of an experienced colleague in their workplace as an ideal learning opportunity and strived to advance their knowledge, practice clinical skills, and broaden their professional experience under his/her supervision. A female nurse with a work experience of seventeen years expressed,

*My clinical learning was discussion-based and resulted from interacting with others. As I wanted to be a good nurse, I searched for skillful and knowledgeable nurses because they can create a halo of changes around themselves—like a fruitful tree whose branches, leaves, and fruits reach others. I’ve never missed such opportunities and have kept in touch with them (P. 9)*.

3.2.1.2 Learning Support

A supportive learning environment heartens learners for learning new things. A female nurse with a fifteen-year work experience said,

*When I started practicing nursing in a dialysis unit, I felt frustrated despite having considerable experience of working in an emergency department. My colleagues supported me and boosted my morale. The unit head-nurse assigned me to an experienced nurse. That nurse, trained and supervised me and gave me constructive feedbacks. I remember that she said to me, ‘Patients receiving dialysis have distended veins and hence, when performing venipuncture, you should put the tip of the needle on the ventral rather than the lateral surface of the vein. Otherwise, the vein will be ruptured’ (P. 4)*.

#### 3.2.2 Masterly Performance

After the apprenticeship step, nurses had strived to independently implement nursing procedures and develop their skills. The two sub-categories of this category were role-modeling and anxiety management.

3.2.2.1 Role-Modeling

The participating nurses had role-modeled their knowledgeable, experienced, and skillful colleagues who had strong motivation for clinical practice as well as good professional skills such as problem-solving. A male nurse having a work experience of three years mentioned,

*I had a knowledgeable and expert colleague who was eager to solve patients’ problems. I had a good feeling about his knowledge and practice. In my opinion, he was a real nurse— knowledgeable, skillful, and interested in managing patients’ problems. Well, I modeled him and gradually became interested in working in the same way as he did (P. 16)*.

3.2.2.2 Anxiety Management

Primarily, our participants did their work-related activities and performed their tasks while having great anxiety. However, they repeatedly performed their activities and tasks to acquire mastery and manage their anxiety. A male nurse with a work experience of three years mentioned,

*Experience is a wonderful thing. I have some levels of fear and anxiety while doing a new clinical task. However, alongside practicing and doing the task for several times, I learn to work more actively (P. 19)*.

Moreover, a female participant having a work experience of fifteen years highlighted,

*Another way for promoting my learning was learning by doing. An instance was the insertion of an NGT. I had read in books that we should first insert the tube into the fossa and once it reaches the oropharynx, we should bend patient’s head and ask him/her to swallow it. However, there was nothing in books about the nasopharynx and managing difficulties in inserting NGT into nasopharynx. I learned about that through using the trial and error method and practicing this skill (P. 10)*.

#### 3.2.3 Self-Regulation

Our participants tried to efficiently use their attitudes, knowledge and expertise in their daily practice. In other words, their performance was mainly self-regulated. This category consisted of three sub-categories of maintaining a caring attitude, having a subjective care plan, and practicing as a prerequisite to expertise.

3.2.3.1 Maintaining a Caring Attitude

According to our participants, self-directed nurses have self-supervision skills and hence, value patient monitoring and provide patient care without needing external supervision. A female participant with a work experience of four years noted,

*I’m very active at work. Excessive fatigue or external supervision does not matter. I know that I should provide care to patients. I search to find patients’ problems and overcome them accordingly. A practice like this gives me such good feelings that I become interested in following it more eagerly (P. 8)*.

Moreover, a male nurse who had a work experience of four years mentioned,

*The more important point I want to say is that a nurse who works in CCU should be on a standby mode. At night shifts that nobody supervised me, I was always looking at monitoring devices. In other words, I had become self-supervised (P. 18)*.

3.2.3.2 Having a Subjective Care Plan

Our participants’ another activity for SDL was having a subjective care plan. They highlighted that in emergency situations they are able to identify patients’ problems and provide holistic care by using a subjective care plan. A female participant who had an eleven-year work experience said,

*A work experience of several years in intensive care units has made my care practice purposeful. Primarily, my practice mainly focused on symptom management. However, currently, when I’m caring for high-risk patients, I try to search and find different ways for managing their problems. For instance, I had a patient with intracranial hemorrhage. Based on a subjective pattern, I understood that this patient has or will develop disorders such as respiratory distress, speech dysfunction, incontinence, confusion, immobility, and impaired nutrition. Accordingly, I focused my care on these disorders (P. 11)*.

3.2.3.3 Practicing as a Prerequisite to Expertise

Our participants noted that practice, seriousness, and active involvement are the essential prerequisites to SDL. A female participant with an eighteen-year work experience expressed,

*One of my good experiences was learning cardiac arrhythmias. This was a complex skill which I learned through attending classes, practicing, and observing. However, this did not suffice. The important point was that I had to learn to immediately manage arrhythmias after diagnosing them. I learned how to manage arrhythmias again by practicing in real situations. It is like learning how to drive. Despite having a driver’s license, you will not become an experienced driver unless you drive in a city (P. 13)*.

## 4. Discussion

We conducted this study to explore Iranian clinical nurses’ activities for SDL. The two main activities of nurses for SDL were striving for knowledge acquisition and striving for skill development. Study findings revealed that participants performed activities such as sensory perception, self-evaluation, and suspended judgment for acquiring and developing their knowledge. These activities are similar to the reflective observation and the abstract conceptualization steps of the Kolb’s Learning Cycle. According to Kolb (1981), reflective observation is a step in which a leaner observes, listens, and assesses subjects and ideas from different perspectives in order to find their meanings. On the other hand, abstract conceptualization is the application of thinking and logic for promoting learning. Planning and analysis are parts of abstract conceptualization. Reflective observation and abstract conceptualization are used for assimilating knowledge and experience (Kolb). Accordingly, study participants’ activities for acquiring and developing their knowledge can be equated with assimilation learning styles reported by Kolb (1981). Assimilating learners combine reflective observation with abstract conceptualization, use inductive learning, and are more interested in abstract concepts than application (de Oliveira et al., 2015).

One of the sub-categories of the striving for knowledge acquisition category was sensory perception. Activities such as careful observation of patients, colleagues, and physicians’ behaviors, active listening, history taking, and participating in nursing and medical rounds were essential to our participants’ learning. The results of a qualitative study conducted in Finland also showed that nursing students promoted their learning by observing different patients, particularly patients with new and rare diseases (Suikkala & Leino-kilpi, 2005). Mackey et al. (2014) also reported observation as a major learning style adopted by nursing students. Moreover, Baraz, Memarian and Vanaki (2014) noted that nursing students carefully observe the performance of their clinical instructors, practicing nurses, and physicians for promoting their learning. All these findings confirm that observational learning is one of nurses’ strategies for acquiring clinical expertise.

Our findings also revealed that nurses’ another activity for SDL was active listening during care provision and ward rounds. Gidman (2013) also noted that care receivers are a valuable source for promoting nurses’ learning and hence, active listening to and effective communication with them can help nurses identify their problems and understand their subjective and emotional viewpoints. Gardner, Woollett, Daly, Richardson, and Aittken (2010) also reported ward rounds as a natural environment for patient-centered learning and evidence-based practice.

Study findings showed that the next step to knowledge acquisition was self-evaluation. Our participants described their previous experiences, identified why and how the events happen, questioned routines, and reflected on their own practice to find more effective alternatives. These findings are consistent with Gibbs Reflective Cycle. According to Gibbs, reflection happens in a cyclic pattern which includes the six steps of description, feelings, evaluation, analysis, conclusion, and action plan (Haghani & Sadeghi, 2012). Bulman, Lathlean and Gobbi (2012) also reported that the steps of reflection are sensible perception of experiences, criticism of experiences, and action as a step to reflection. Accordingly, the art of self-evaluation encourages nurses to seek solution for complicated situations during their clinical practice and finally gives them a sense of possession over the acquired knowledge. Moreover, self-evaluation is a means for promoting nurses’ critical thinking and clinical judgment (Lasater, 2011) and prepares them for the next step of knowledge acquisition, i.e. suspended judgment.

Suspended judgment was another activity performed by the study participants for SDL. The complexity of patients’ conditions as well as nurses’ professional experience affected their information processing, critical thinking, and suspended judgment. In other words, nurses’ critical thinking was focused on screening, early risk identification, and hypothesis making. These findings are in line with the findings reported by Javadi, Paryad, Roshan, Fadakar and Asiri (2011). They found that nursing students processed information step-by-step to decide upon the likelihood of risks. Kuiper, Pesut and Kautz (2009) also noted that for effective patient management in emergency situations, nursing students need to have a general picture of potential risks, relevant nursing diagnoses, and care plans. Consequently, critical thinking is among the most effective learning strategies and helps nurses make accurate clinical judgments.

The second main category of the study was striving for skill development. Study participants performed activities such as apprenticeship, masterly performance, and self-evaluation for developing their clinical skills. These findings are in agreement with the concrete experience and the active experimentation steps of the Kolb’s Learning Cycle (Kolb, 1981). In other words, our participants used the accommodation learning styles reported by Kolb (1981). Concrete experience denotes active involvement, interpersonal communication, and learning through experiences while active experimentation necessitates engaging in the implementation of plans. Accommodating learners are excellent in doing activities and like new experiences (El-Gilany & Abusaad, 2013).

One of the activities performed by the participating nurses for skill development was apprenticeship which implies learning from expert colleagues in a supportive learning environment. Freeling and Parker (2015) also reported that new graduate nurses are not qualified enough for clinical practice and hence, they need to develop their clinical skills through apprenticeship. The findings of a qualitative study done by Eller, Lev and Feurer (2014) also revealed that mutual respect and supportive feedbacks were among the key elements of apprenticeship programs. In apprenticeship programs, junior nurses imitate experienced colleagues and seek knowledge from them. Apprenticeship improves nurses’ performance and broadens their professional experience. Carlson (2014) also noted that apprenticeship programs gives rise to critical yet friendly colleagues who evaluate each others’ performance while maintaining friendship and mutual trust.

Another activity performed by our participants for developing their skills and promoting their learning was masterly performance which implies role-modeling for better clinical performance. Myrick, Yonge and Billay (2010) also noted that as a requisite skill to ethical patient care, good and punctual practice is acquired through the process of role-modeling. Moreover, the findings of a review study conducted by Baldwin, Mills, Birks, and Budden (2014) indicated that nursing students role-modeled experienced nurses for transferring their knowledge to practice and demonstrating professionalism. Accordingly, nurses need to observe and model experienced role-models’ behaviors and receive constructive feedbacks from them for promoting their learning and acquiring professional competence.

After learning from role-models, our participants strived to practice the learned skills in order to manage their anxiety, promote their learning, and improve their clinical performance. Öztürk, Caliskan, Gocmen Baykara, Karadag and Karabulut (2015) also reported a similar finding. They conducted a four-year interventional study to evaluate the effects of regular trainings on nursing students’ basic psychomotor skills and found that after their intervention, students were more competent in doing nursing procedures learned in the first year due to practicing the procedures for many times. Moreover, students who were incompetent in doing some procedures had strived to acquire competence by doing those procedures repeatedly.

The final activity performed by our participants for SDL was self-regulation which entails effective use of attitudes, beliefs, and skills in daily clinical practice. The sub-categories of this category were maintaining a caring attitude, having a subjective care plan, and practicing for gaining expertise. Our findings revealed that self-regulation promoted nurses’ self-supervision and self-evaluation. Chen, Stocker, Wang, Chung and Chen (2009) also found that nursing students who actively participated in self-regulation activities had more positive attitude towards and deeper pleasure at doing learning activities. Moreover, they found that students gradually developed self-supervision and independence through attending continuing education programs and using the trial and error technique.

### 4.1 Limitations

In this qualitative study, we explored a group of Iranian nurses’ activities for SDL. Despite attempting to recruit a maximum variation sample, the study findings cannot be widely transferred to other settings and populations. Further studies are needed for exploring nurses’ activities for SDL in other countries and contexts.

## 5. Conclusion

Study findings suggest that Iranian clinical nurses perform different self-directed intellectual and experiential activities for promoting their learning. Nurses continually revise their personal performance by performing self-directed intellectual and experiential activities in order to acquire expertise. The process of acquiring expertise is a linear process which includes two key steps of knowledge acquisition and knowledge development. To acquire and advance their knowledge, nurses perform mental learning activities such as sensory perception, self-evaluation, and suspended judgment step-by-step. On the other hand, they develop their skills through doing activities like apprenticeship, masterly performance, and self-regulation. At any given time, a nurse is on different points of this expertise acquisition process. In other words, she/he may be in the knowledge acquisition step for learning skill A and at the same time, in the skill development step for skill B. The absolute prerequisite to expertise acquisition is that a nurse needs to follow these two steps in a sequential manner.
